# Vertebral artery dissection presenting with ispilateral acute C5 and C6 sensorimotor radiculopathy: A case report

**DOI:** 10.1186/1757-1626-1-139

**Published:** 2008-09-03

**Authors:** Ghazaleh Tabatabai, Wolfgang Schöber, Ulrike Ernemann, Michael Weller, Rejko Krüger

**Affiliations:** 1 Center of Neurology and Hertie Institute for Clinical Brain Research, University of Tübingen, Germany; 2 Department of Neuroradiology, University of Tübingen, Germany

## Abstract

Spinal manifestations of vertebral artery dissection (VAD) are rare events and are typically symptomatic with neck pain and ischemic brain injury. We report a patient presenting with unusual peripheral paresis of the right upper limb due to an intramural hematoma of the right vertebral artery with local compression of C5 and C6 as the cause of cervical radiculopathy. These symptoms completely resolved after anticoagulation and physical therapy.

## Case presentation

A 51-year-old women presented with a two-weeks history of progressive pain and weakness of her right arm. Ten days earlier an orthopaedic specialist had performed a chiropractic maneuver to resolve the pain radiating from the neck to the right upper arm without alleviation of the symptoms. Four days later, the patient had noticed a weakness of elevation and flexion of her right upper arm. A cervical spine MRI scan displayed mild protrusion of the disc between the fifth and sixth cervical vertebrae resulting in a mild compression of the right C6 root and a surgical intervention due to progressive and substantial weakness was considered.

**Figure 1:**
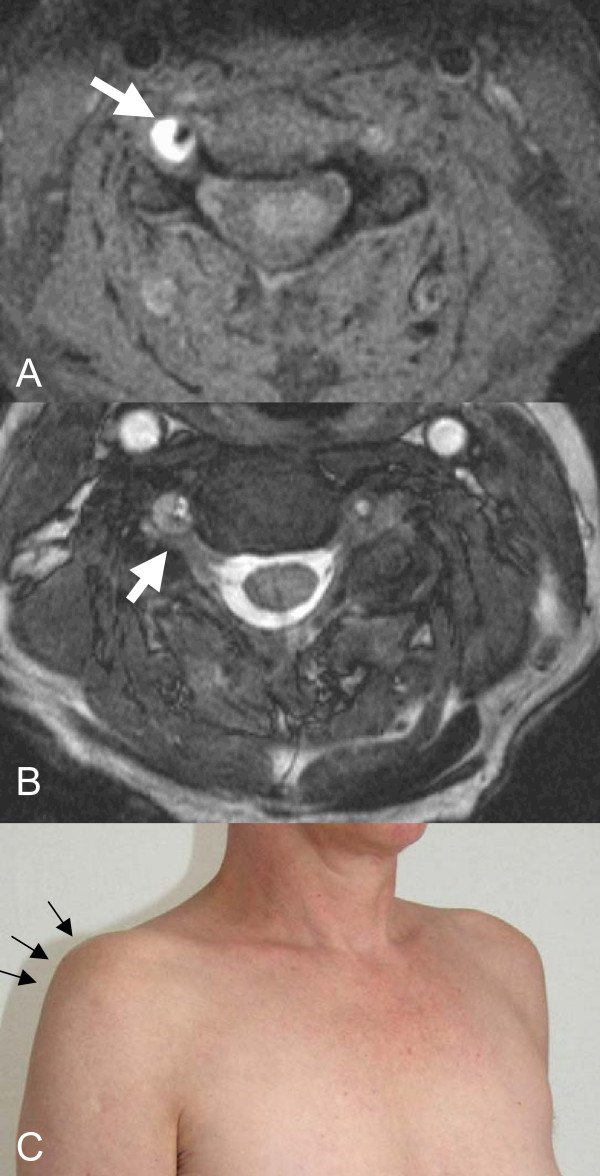
**MRI of the cervical spine, axial views (A,B): The T1-weighted fat-saturated axial image (A) shows an eccentric hyperintensity surrounding the right VA at C5 level (arrow) indicating an intramural hematoma.** The T2-weighted image **(B)** indicates compression of spinal root C5 (arrow) by the dissecting aneurysm.

Examination at admission revealed a distribution of the pain along the dermatomes C5 and C6, a paralysis of the deltoid (0/5) and the biceps brachii muscle (2/5), an atrophy of both muscles, decreased biceps and deltoid reflexes as well as sensory deficits in the right C5 and C6 dermatomes. However, the available MRI scan did not explain the more pronounced affection of the C5 root. MRI angiography then revealed a right vertebral artery dissection with a dissecting aneurysm with maximum expansion at the C5 level. The anterolateral part of the C5 and C6 radixes were compressed, causing cervical radiculopathy at these levels (Fig. 1). The MRI scan of the brain displayed no signs of ischemic insult or other pathologies. Due to hypoplasia of the contralateral vertebral artery, a surgical intervention was not taken into account. Thus a conservative approach with oral warfarin administration and a symptomatic treatment for cervical radiculopathy was started.

After eight weeks of anticoagulation and physical therapy, the weakness of the upper arm markedly improved with an improvement in muscle strength of the deltoid (4/5) and the biceps brachii (5/5) without any sensory deficits. The follow-up cervical MRI scans showed a regularly revascularized right vertebral artery. Four months later, muscle strength of the deltoid completely recovered.

## Discussion

Spinal manifestations of vertebral artery dissection (VAD) are a rare event.[1] Typical manifestations of VAD include brain ischemia combined with neck pain.[2] In previous reports of patients with spinal manifestations of VAD the prominent sign was spinal ischemic cervical myelopathy.[2-4] Recently a rare manifestation of VAD with left shoulder weakness and numbness of the left upper limb was reported that was successfully treated by proximal occlusion of the dissected vertebral artery using detachable balloon and Guglielmi detachable coils.[5] Proximal vertebral artery occlusion using an intravascular technique was regarded as a non-invasive and effective option for patients with a cervical radiculopathy due to cervical vertebral artery dissection. Here we report local compression due to expansion of the dissecting aneurysm as a mechanism for sensorimotor radiculopathy with a favorable outcome after anticoagulation and physical therapy. Therefore VAD may be considered as a cause of otherwise unexplained cervical radiculopathic symptoms.

## Competing interests

The authors declare that they have no competing interests.

## Authors' contributions

GT, MW and RK analyzed and interpreted the patient data regarding the neurological symptoms and the management of the patient. GT performed the neurological examination and the follow up examinations. GT and RK wrote the manuscript. WS and UE analyzed and interpreted the MRI. All authors read and approved the final manuscript.

## Consent

Written informed consent was obtained from the patient for publication of this case report and accompanying images. A copy of the written consent is available for review by the Editor-in-Chief of this journal.
